# Development of Corn Starch-Neusilin UFL2 Conjugate as Tablet Superdisintegrant: Formulation and Evaluation of Fast Disintegrating Tablets

**DOI:** 10.1155/2014/827035

**Published:** 2014-09-23

**Authors:** Prateek Juneja, Birender Kaur, Oluwatoyin A. Odeku, Inderbir Singh

**Affiliations:** ^1^Chitkara College of Pharmacy, Chitkara University, Chandigarh-Patiala National Highway, Rajpura, Patiala, Punjab 140401, India; ^2^Department of Pharmaceutics & Industrial Pharmacy, Faculty of Pharmacy, University of Ibadan, Ibadan 200005, Nigeria

## Abstract

In the present study, corn Starch-Neusilin UFL2 conjugates were prepared by physical, chemical, and microwave methods with the aim of using the conjugates as tablet superdisintegrant. Various powder tests, namely, angle of repose, bulk density, tapped density, Hausner's ratio, Carr's index, swelling index, and powder porosity were conducted on the samples. The conjugates were characterized by ATR-FTIR, XRD, DSC, and SEM techniques. Heckel and Kawakita models were applied to carry out compression studies for the prepared conjugates. Fast disintegrating tablets of domperidone were prepared using corn starch and corn Starch-Neusilin UFL2 conjugates as tablet superdisintegrants in different concentrations. Conjugates were found to possess good powder flow and tabletting properties. Heckel analysis indicated that the conjugates prepared by microwave method showed the slowest onset of plastic deformation while Kawakita analysis indicated that the conjugates prepared by microwave method exhibited the highest amount of total plastic deformation. The study revealed that the corn Starch-Neusilin UFL2 conjugates possess improved powder flow properties and could be a promising superdisintegrant for preparing fast disintegrating tablet. Also, the results sugessted that the microwave method was found to be most effective for the preparation of corn Starch-Neusilin UFL2 conjugates.

## 1. Introduction

Amongst innumerable applications, starch and its derivatives are one of the most widely used excipients in the pharmaceutical industry as they are incorporated in the manufacture of assorted dosage forms due to their biodegradability and biocompatibility. Starches have become a valuable ingredient because of their inertness, abundance, and cost effectiveness in the food industry, where they are used as thickeners, bulking, water retention, and gelling agents, in the pharmaceutical industry where they are used as fillers, binders, and disintegrants in tablet formulations. Commercially, starches are obtained from a variety of cereals (corn, waxy corn, high amylose corn, wheat, and various rice varieties) and from the tubers and roots (predominantly potato and cassava). In the pharmaceutical formulations, starch can be used both as a disintegrant and as a binder depending on the explicit attributes crucial for the formulation. As a disintegrant, the mechanism of action of starch includes wicking, the imbibitions of water into the tablet matrix via capillary action. The concentration of starch used is crucial; if it is below the optimum concentration then there are insufficient channels for capillary action and if it is above the optimum concentration then it is difficult to compress the compacts. Generally, starch can be used at a concentration range of 2–10% w/w. As a binding agent in tablet formulations, starch is heated in solution enhancing its conversion into a paste before addition to the formula powder blend [1, 2]. Disintegrants are essential components of tablet formulations since tablet disintegration is a prerequisite for dissolution of the active drug from the tablet. Starch is one of the earliest known disintegrants although, in recent times, super disintegrants such as sodium starch glycolate and croscarmellose sodium offer significant improvements over the native starches. Physical and chemical modifications have been used to improve the compaction properties of some native starches and have yielded starches with better disintegration properties and some have been found to be useful for sustained release dosage forms [3–5]. Rashid et al. [1] developed a directly compressible excipient by coprocessing starch with magnesium silicate which was achieved either by coprecipitation of magnesium silicate onto different types of starch or by dry granulation of maize starch with magnesium silicate. Magnesium silicate, as an antiadhering agent, increased the permeability of both maize and partially pregelatinized starch, resulting in compacts of high mechanical strength, short disintegration time, and low lubricant sensitivity. Formulation with this novel excipient system, using paracetamol as a model drug, indicated its suitability as a single multifunctional excipient. Staroszczyk [6] performed silication of potato starch by microwave irradiation. It was reported that while increasing the silicating agent cross-linking of starch was increased and silicated starches are thermally more stable. A number of modification techniques, namely, physical, chemical, enzymatic, and genetic, have been reported with an aim to enhance the positive attributes and eliminate short coming of the native starches [7].

In recent times, advancement in budding new excipients with multifunctional capabilities (as glidants, fillers, binders, and disintegrants) has exploited the use of silicates produced synthetically. Silication of excipients resulted in improvement in various properties of the materials, namely, mechanical strength, compressibility, disintegration, and sensitivity towards lubrication. Thus silicate/starch preparations have the potential to fulfil the requirements of a multifunctional excipient. In this context the physical properties of compacts produced (disintegration time, tensile strength, and powder compressibility) and sensitivity towards lubrication need to be investigated. On this basis, the current study reports the application of silicates as an effective tabletting aid when used with corn starch [8, 9].

Neusilin is a synthetic, amorphous form of magnesium aluminometasilicate which can be used as a multifunctional excipient in both direct compression and wet granulation of solid dosage forms. Neusilin is widely used in improving the quality of tablets (in the terms of its compaction and disintegration properties), powders, granules, and capsules. It does not develop gels with aqueous solutions unlike other magnesium aluminium silicates. The different grades of Neusilin available are alkaline (FH1, FH2, FL1, FL2, S1, S2, and SG2) and neutral (US2, UFL2, NFL2N, and NS2N) [10].

The aim of the present study is to evaluate the efficiency of a new conjugate of corn starch and Neusilin, as super disintegrating agent in the tablets. The corn Starch-Neusilin UFL2 conjugates were prepared by three different methods, namely physical, chemical, and microwave, and were used as a superdisintegrant in domperidone tablet formulation and the tablet properties were compared with a standard marketed fast disintegrating tablet. Domperidone (C_22_H_24_ClN_5_O_2_, MW-425.91), poorly water-soluble drug [11], is a potent antidopaminergic drug used orally and intravenously for suppressing nausea and vomiting. An adult dose of 10 mg domperidone has a modest activity without extrapyramidal side effects as it crosses blood brain barrier poorly. Domperidone is absorbed orally and its metabolites are completely biotransformed and excreted in urine. The dissolution rate and bioavailability of a poorly soluble drug from solid dosage form depend much on formulation additives and formulation characteristics.

## 2. Materials and Methods

Commercial corn starch was supplied by IPHZA Pharmaceuticals, Patiala, Punjab, India. Neusilin UFL2 (Fuji Chemicals, Japan) was supplied as gift sample by Gangwal Chemicals, Mumbai, India. Domperidone was gifted by Dr. Reddy's Laboratories, Baddi (H.P), India. NaOH was procured from Merck Specialities Pvt. Ltd., Mumbai, India. All reagents used were of analytical grade.

### 2.1. Preparation of Corn Starch-Neusilin UFL2 Conjugates

Three methods were used in the formulation of the corn Starch-Neusilin UFL2 conjugate: physical, chemical, and microwave methods. The* physical method* involved simple mixing of corn starch and Neusilin UFL2 in the ratio 1 : 1. To ensure proper mixing tumbling method was used and the weighed starch and Neusilin were transferred into a beaker and the mouth of the beaker was closed ensuring that there is no spillage of the contents from the beaker while the mixing is carried out. The mixing was carried out for 15 minutes. In* chemical method* corn starch and Neusilin UFL2 were incorporated in the ratio 1 : 1. Corn starch was suspended in distilled water (q.s) and kept on magnetic stirrer at 270 rpm and temperature not more than 40°C. To this, solution of Neusilin UFL2 in NaOH (q.s) was gradually added with constant stirring. After 15–20 minutes the precipitated conjugate was filtered using Whatman filter paper (GE Healthcare UK Limited) having a pore size of 125 mm and dried in oven at a temperature not more than 50 ± 2°C. In* microwave method* physical mixture of corn starch and Neusilin UFL2 was subjected to microwave radiation at 590 watt for not more than 5 minutes.

### 2.2. Formulation of Fast Disintegration Tablets

Fast disintegrating tablets of domperidone (10 mg) were formulated by direct compression method using corn starch and the corn Starch-Neusilin UFL2 conjugates as superdisintegrant, according to the formulae given in Table 1. The specified quantity of the drug, diluent, and corn starch or corn Starch-Neusilin UFL2 conjugates were weighed, accurately, passed through 60 mesh sieves (250 *μ*m opening size), and mixed by tumbling method for 15 to 20 min (contents were transferred into a beaker and the mouth of the beaker was closed ensuring that there is no spillage of the contents from the beaker while mixing is carried out). The blend was lubricated with talc and magnesium stearate and mixing was done for additional 5 minutes and the resulting powder mixture was compressed into tablets using multipunch tabletting machine (AK Industries, Nakodar, Punjab, India) using 6.75 mm biconcave round die-punches. The compression force was adjusted to give tablet hardness in the pharmacopoeial range of fast disintegrating tablets (3–5 kg/cm^2^). For each batch 200 tablets were formulated.

### 2.3. Precompression Evaluation

#### 2.3.1. Angle of Repose (*θ*°)

The angle of repose of powder blends was determined by the funnel method. Accurately weighed powder blends were taken in a funnel. The height of the funnel was adjusted in such a way that the tip of the funnel just touched the apex of the heap of the powder blends (2 cm). The powder blends were allowed to flow through the funnel freely onto its surface. The diameter of the powder cone was measured and angle of repose was calculated [12]. Three determinations were performed.

#### 2.3.2. Bulk Density and Tapped Density

Both loose bulk density (LBD) and tapped bulk density (TBD) were determined. A quantity of 1 gm of powder was introduced into a 10 mL measuring cylinder. After the initial volume was determined, the cylinder was tapped onto a hard surface from the height of 2.5 cm at 2 seconds intervals. Tapping was continued until no further change in volume was noted. The LBD and TBD were calculated [12]. The determination was carried out in triplicate.

#### 2.3.3. Compressibility Index and Hausner Ratio

The compressibility index of the powder blends was determined by Carr's compressibility index or Carr's index (CI). Hausner ratio (HR) was also determined for each powder blend [12].

#### 2.3.4. Swelling Index

Initial bulk volume of the powder was evaluated using 100 mL stoppered graduated cylinder. Water was added in sufficient quantity to produce uniform dispersion. The sediment volume of the swollen mass was measured after 24 hours. The swelling index was calculated as

(1) $$\begin{array}{c} \text{Swelling}\;\text{index}=\left(V_{2}-\frac{V_{1}}{V_{1}}\right)*100, \end{array}$$

where *V*_1_ and *V*_2_ are initial volumes of the powder before and after hydration, respectively.

#### 2.3.5. pH

Dispersion (1% w/v) of the sample was prepared in distilled water and the pH was determined individually using digital pH meter at 37 ± 2°C.

#### 2.3.6. Loss on Drying

Loss on drying (LOD) is used to determine the levels of moisture or solvents present in the sample. The sample was weighed (*W*_1_) and heated in an oven at 100 ± 5°C for 2 hrs. Sample was cooled in the dry atmosphere of a desiccator and then reweighed (*W*_2_). %LOD was calculated by

(2) $$\begin{array}{c} \%\text{LOD}=\left(W_{1}-\frac{W_{2}}{W_{1}}\right)*100. \end{array}$$

#### 2.3.7. Effective Pore Radius (*R*_*eff*.*P*_)

Effective pore radius of the powder was determined using method reported by Goel et al. [13]. A micropipette tip (2 mL, transparent) was completely filled with powder and weighed (*W*_*i*_). Then *n*-hexane (surface tension (*γ*) is 18.4 mN/m) was poured drop wise on bed top till the solvent filtered out at the bottom of the tip. The tip was reweighed (*W*_*f*_) and effective pore radius was calculated by

(3) $$\begin{array}{c} R_{\text{eff}.\text{P}}=W_{f}-\frac{W_{i}}{2\pi \gamma }\text{.} \end{array}$$

### 2.4. Attenuated Total Reflectance-Fourier Transform IR Spectroscopy (ATR-FTIR)

The infrared (IR) spectra of samples were obtained using an Attenuated Total Reflectance-Fourier Transform Infra-Red (ATR-FTIR) spectrophotometer (Alpha, Bruker, Japan). The samples were scanned in the spectral region of 4000 cm^−1^ to 400 cm^−1^ by KBr pellet method.

### 2.5. X-Ray Powder Diffraction (XRPD)

The X-ray powder diffractograms were registered in an X-Pert Pro (USA) in Bragg-Brentano geometry, using glass tubing with a Cu anode and graphite monochromator. The diffractometer was operated at 40 mA and 40 kV. All the samples being a size of less than 250 *μ*m were randomly placed on a glass slide, respectively. The signals of the reflection angle of 2*θ* were recorded from 0° to 60° at a scanning rate 0.21°/sec.

### 2.6. Scanning Electron Microscopy (SEM)

The scanning electron micrographs were taken to study the surface morphology using a Hitachi (Model S 4300 SE/N SEM, Hitachi High Technologies, Singapore) at an accelerator potential of 10 kV. The samples were stuck on a specimen holder using a silver plate and then coated with palladium in a vacuum evaporator.

### 2.7. Differential Scanning Calorimetry (DSC)

The thermal transitions of starch samples were investigated with the use of a Perkin Elmer DSC 8000 apparatus (USA), calibrated by using a high purity indium standard. Samples about 10 mg were hermetically sealed in flat bottomed aluminium pans and heated in an atmosphere of nitrogen to eliminate the oxidative and pyrolytic effects. The heating rate was 5°C/min in a temperature range of 25–300°C. The DSC thermograms were recorded.

### 2.8. Compression Study

The two well-known compression models The Heckel and The Kawakita were used to carry out the studies [14].

#### 2.8.1. Determination of Precompression Density

The bulk density of each sample at zero pressure was determined by pouring the granules through a funnel into a glass measuring cylinder with a 24 mm diameter and a volume of 50 mL at an angle of 45°. Determinations were done in triplicate. The relative density, *D*_0_, of each formulation was obtained from the ratio of its bulk density to its particle density [15].

#### 2.8.2. Preparation of Tablets for Compression Studies

Tablets (400 mg) were prepared by compressing the prepared conjugates for 30 seconds with predetermined loads on a hydraulic press (Model CAP15T-1233, PCI Analytics Pvt. Ltd., Mumbai, India). After ejection, the tablets were stored over silica gel for 24 hours. Their masses (*m*) and dimensions were then determined, respectively, and their relative densities (*R*) were calculated using the equation

(4) $$\begin{array}{c} R\;=\frac{m}{V_{t}\rho_{s}}, \end{array}$$

where *V*_*t*_ is the volume (cm^3^) of the tablet and *ρ*_*s*_ is the particle density (g/cm^3^) of the solid material [15].

#### 2.8.3. The Heckel Function

The deformation mechanism of the material was determined using the Heckel model, as it is widely used for relating the relative density, *D*, of a powder bed during compression to the applied pressure, *P*. The displacement and force data, registered on the simulator, were transferred to a spreadsheet program (Microsoft Excel 2007). The force values were converted into pressures and the displacement values (in combination with the individual tablet weight determined immediately after compaction) and the true density of the material (determined before compaction) were used to calculate the apparent density of the powder bed, 1/(1 − *D*). Using the Heckel plot, the compression stage was subjected to linear regression analysis. It is denoted as

(5) $$\begin{array}{c} \ln\left[\frac{1}{\left(1-D\right)}\right]=KP+A. \end{array}$$

The slope of the straight line portion, *K*, is the reciprocal of the mean yield pressure, *P*_*y*_, of the material. From the intercept *A*, the relative density, *D*_*A*_, can be calculated using the following equation:

(6) $$\begin{array}{c} D_{A}=1-e^{-A}. \end{array}$$

Relative density of the powder at the point when the applied pressure equals zero, *D*_0_, is used to describe the initial rearrangement phase of densification as a result of die filling;

(7) $$\begin{array}{c} D_{B}=D_{A}-D_{0}. \end{array}$$

Relative density, *D*_*B*_, describes the phase of rearrangement at low pressures and is the difference between *D*_*A*_ and *D*_0_ [15, 16].

#### 2.8.4. The Kawakita Function

In the Kawakita equation, the particle density is not introduced in the calculations since the model operates on the relative change in volume which gives the same result whether the relative or the absolute volume is used. The problem in calculation of this equation is to find the correct initial volume, *V*_0_. The Kawakita equation is used to study powder compression using the degree of volume reduction (*C*) and is written as

(8) $$\begin{array}{c} C=\frac{\left(V_{0}-V_{p}\right)}{V_{0}}=\frac{abP}{\left(1+bP\right)}\text{.} \end{array}$$

The equation, in practice, can be rearranged to give

(9) $$\begin{array}{c} \frac{P}{C}=\frac{P}{a}+\frac{1}{ab}, \end{array}$$

where *V*_0_ is the initial bulk volume of the powder and *V*_*p*_ is the bulk volume after compression. Constant “*a*” is equal to the minimum porosity of the material before compression while constant “*b*” is related to the plasticity of the material. The reciprocal of “*b*” gives the pressure term, *P*_*k*_, which is the pressure required to reduce the powder bed by 50% [14, 15].

### 2.9. Postcompression Evaluation

#### 2.9.1. Diameter and Thickness

A calibrated vernier calliper (M/s Mitutoyo Corp., Japan) was used for diameter and thickness evaluation of tablets.

#### 2.9.2. Hardness

The tablet hardness is the force required to break a tablet in a diametric compression force. Hardness tester (Perfit, India) was used to determine the force required to break the tablet diametrically. The test was performed on six tablets and the average was calculated.

#### 2.9.3. Friability

The friability (*F*) of a sample of 20 tablets was measured using Roche Friabilator (Model 902, EI, Panchkula, India). Twenty tablets were weighed and rotated at 25 rpm for 4 min. Tablets were reweighed after removal of fines (dusted) and the percentage of weight loss was calculated. Friability below 1% was considered acceptable.

#### 2.9.4. Weight Variation Test

Weight variation test was done by weighing 20 tablets, individually, calculating the average weight, and comparing the individual tablet weight to the average weight.

#### 2.9.5. Content Uniformity

The content uniformity was assessed according to USP method. Ten tablets were pulverized and quantity of powder equivalent to 10 mg of domperidone was shaken with 100 mL of 0.1 N HCl for 30 minutes. The contents were filtered through a 0.45 *μ*m membrane filter, diluted, and analyzed at 284 nm using a UV/VIS double beam spectrophotometer (Model 2202, Systronics, Ahmedabad, India) [17].

#### 2.9.6. Wetting Time

A piece of tissue paper (10.75 × 12 mm) folded twice was placed in a culture dish (*d* = 6.5 cm) containing 6 mL of water (containing a water soluble dye eosin). A tablet was carefully placed on the surface of tissue paper and the time required for water to reach the upper surface of the tablet was noted as the wetting time [18].

#### 2.9.7. Water Absorption Ratio

The same procedure used for the wetting time was used for the water absorption ratio. Initial weight of the tablet (*W*_*a*_) was measured before its placement on the wet tissue and the final weight (*W*_*b*_) was taken after complete wetting. Water absorption ratio, *R*, was determined using the equation [19]

(10) $$\begin{array}{c} R=100\;\frac{\left(W_{b}-W_{a}\right)}{W_{b}}\text{.} \end{array}$$

#### 2.9.8. Porosity

Porosity is a measure of the void spaces in a material and is a fraction of the volume of voids over the total volume, between 0 and 1, or as a percentage between 0 and 100 percent. The porosity of the tablets was calculated as follows:

(11) $$\begin{array}{c} \text{Porosity}=\frac{1-m}{\rho_{\text{true}}}*V, \end{array}$$

where *ρ*_true_ is the true density of the mixture and *m* and *V* are the weight and volume of the tablet, respectively. The true density of the powder was found using true density meter (SMART PYCNO 30, Smart Instruments, Maharashtra, India).

#### 2.9.9. Tablet Packing Fraction (*P*_*f*_)

The tablet packing fraction, *P*_*f*_, is a measure of the degree of consolidation/compactness of the tablet. Tablet packing fraction was determined by the equation

(12) $$\begin{array}{c} \text{Packing}\;\text{fraction}\left(P_{f}\right)=\frac{w}{\pi r2t\rho }, \end{array}$$

where *w* is the weight of a tablet, *r* is radius, *t* is thickness, and *ρ* is the particle density.

The radius and thickness of 10 tablets were measured using a vernier calliper. The apparent particle density of the drug powder was determined using liquid paraffin displacement method. Firstly, the weight of a specific gravity bottle filled with liquid paraffin and the weight of the specific gravity bottle containing a sample of the drug powder were noted. The final weight was determined. The determination was executed in triplicate, and mean results were used in the calculation of *P*_*f*_ [20].

#### 2.9.10. *In Vitro* Disintegration Time

*In vitro* disintegration time for the tablets was determined using USP disintegration apparatus (EI Product, Panchkula, India) using 0.1 N HCl (pH 1-2, 900 mL at 37°C) as the disintegrating medium.

#### 2.9.11. *In Vitro* Dissolution Studies

*In vitro* dissolution of the fast disintegrating tablets was studied in USP XXIV dissolution apparatus II (DS 8000, Lab India, Pune, India) employing a paddle stirrer at 50 rpm using 900 mL of pH 1.2 0.1 N HCl at 37 ± 0.5°C as a dissolution medium. Aliquots of 5 mL each were withdrawn at predetermined time intervals and replaced with equal volume of fresh medium. Aliquots were filtered through a 0.45 *μ*m membrane filter and analyzed for drug content using a UV/VIS double beam spectrophotometer (2202, Systronics, Ahmedabad, India) at 284 nm. Drug concentration was calculated and expressed as cumulative percent of the drug released. The similarity factor (*f*_2_) is a logarithmic transformation of the sum-squared error of differences between the test (*T*_*j*_) and reference (*R*_*j*_) products over all time points. It is a useful tool for comparison of dissolution profiles when more than three or four dissolution time points are available. It is calculated as

(13) f(2)=50×log⁡⁡{[1+(1n)∑j=1nWj|Rj−Tj|  2]−0.5×100}.

(*W*_*j*_) is an optional weight factor. The similarity factor fits result between 0 and 100. It is 100 when the test and reference profiles are identical and tends to be 0 as the dissimilarity increases. In order to consider similar dissolution profiles, *f*_2_ values should be close to 100.

## 3. Result and Discussion

### 3.1. Precompression Evaluation

#### 3.1.1. Micromeritics Study

The result of the micromeritic studies for corn starch and the prepared corn Starch-Neusilin UFL2 conjugates were conducted and results are listed in Table 2. Angle of repose (*θ*°) is a characteristic of the internal friction or cohesion of the particles. Its value will be high if the powder is cohesive and low if the powder is non-cohesive. Flow property of the corn starch was improved by the addition of Neusilin UFL2 as compared formulations with conjugates showed good to excellent flow properties as indicated by the values of angle of repose (36.69–27.67°) to the corn starch (43.25°). Carr's index showed values 22.5 to 12.5 denoting that these formulations were of acceptable to good flowability compared to the corn starch (34.28). Hausner's ratio showed that powders, with low interparticle friction, had ratios of approximately 1.29 to 1.14, indicating good flow properties as compared to the corn starch (1.52). Swelling index of the conjugates prepared by physical, chemical, and microwave method were found to be 40, 65, and 95%, respectively, which was far better than the swelling index of corn starch, that is, 18%, which is in context with the effective pore radius of the conjugates and corn starch. The results of both swelling and effective pore radius point towards more wicking action capability and hence disintegration potential of the conjugates over the pure corn starch. The result of micromeritic study indicates that conjugates prepared by microwave method were the most effective method followed by chemical and physical method as they gave better micromeritic properties as evidenced from the Table 2.

#### 3.1.2. ATR-FTIR Analysis

**Figure 1:**
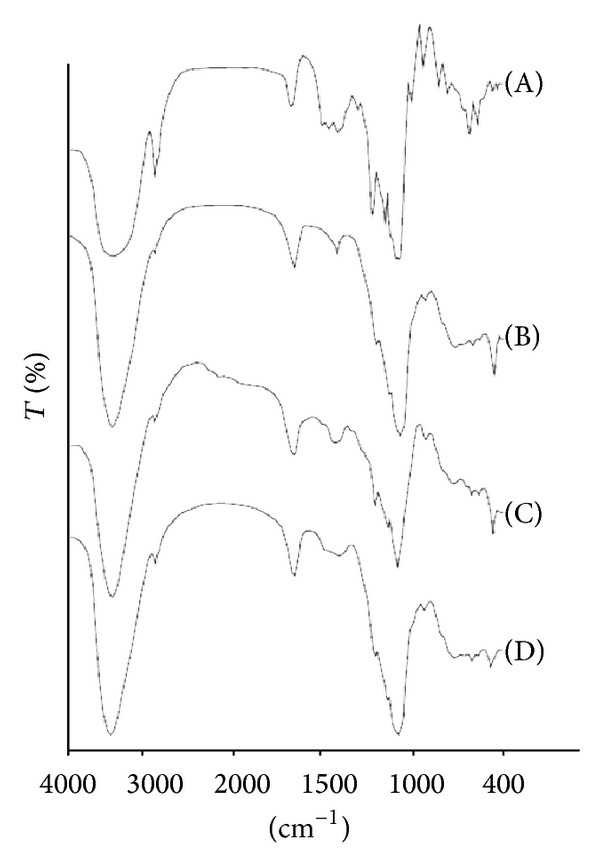
IR spectra of (A) native corn starch; corn Starch-Neusilin UFL2 conjugates by (B) physical method, (C) chemical method, and (D) microwave method.

Corn starch and Neusilin UFL2 interactions studies were carried out using ATR-FTIR spectrophotometer and the spectra for the samples are shown in Figure 1. The IR spectra of corn starch showed a peak at 3434 cm^−1^ and 2931 cm^−1^ representing O–H and C–H stretching, respectively. The absorption band at 1652 cm^−1^ is due to absorbed water in amorphous region of starch. Peak at 1241 cm^−1^ represents CH_2_OH group whereas peak at 1159 cm^−1^ represents coupling mode of C–C and C–O stretching vibrations. The band at 1080 cm^−1^ represents C–O–H bending vibration whereas peak at 929 cm^−1^ could be ascribed to the skeletal mode vibration of α-1,4-glycosidic linkage. The corn Starch-Neusilin UFL2 conjugates prepared by different methods, namely physical, microwave, and chemical, exhibit a sharp peak near 3480 cm^−1^ which is otherwise observed at 3434 cm^−1^ in the pure corn starch. This shift and sharpening of peak indicate the formation of Si–O–C bridging bond between corn starch and Neusilin UFL2. Moreover reduction in the intensity of peak at 1241 cm^−1^, 1159 cm^−1^, and 1080 cm^−1^ confirms intermolecular bridging between corn starch and Neusilin UFL2. Thus, the addition of Neusilin changed the properties of corn starch forming a new excipient with different functional properties.

#### 3.1.3. XRD Analysis

**Figure 2:**
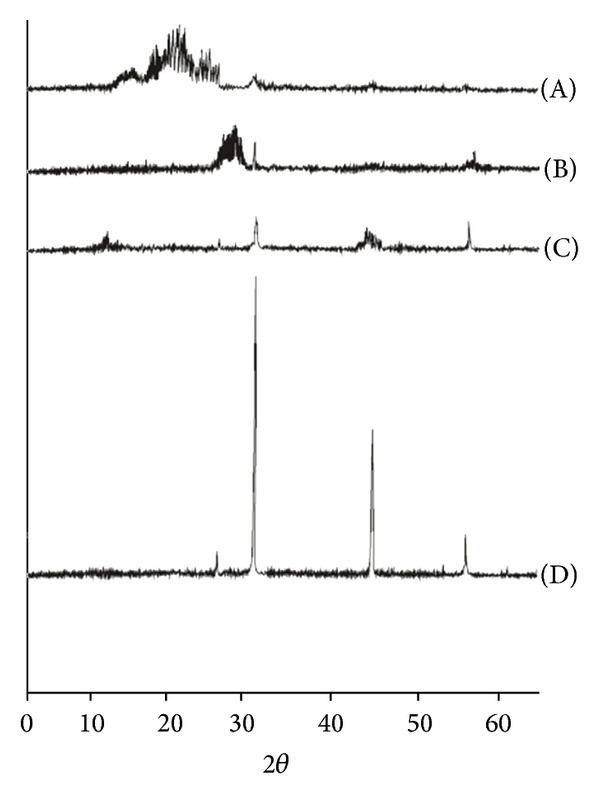
XRD pattern of (A) native corn starch; corn Starch-Neusilin UFL2 conjugates by (B) physical method, (C) chemical method, and (D) microwave method.

The XRD patterns of the samples are shown in Figure 2. The structure of corn starch is characterized by the presence of broad peak at 24.56°2*θ* angle. Appearance of sharp peak at 27.40°2*θ*, 31.72°2*θ*, 45.60°2*θ*, 53.91°2*θ*, and 56.47°2*θ* angles, respectively, in case of conjugates prepared by microwave method gives clear indication of reduction in amorphous nature or otherwise increase in the crystalline behavior of the corn starch that could be correlated with the increase in swelling and hence superdisintegrant potential of conjugates prepared by different methods. Crystallinity and hence the superdisintegrant property (Table 4) of conjugates prepared by different methods were in the rank order of microwave > chemical > physical mixture. High proportion of longer chains might form more stable crystallites in the pure corn starch. The swelling power of corn starch depends on the water holding capacity of the starch molecule by hydrogen bonding. Hence higher degree of intermolecular bonding between corn starch and Neusilin UFL2 favors the long chain crystalline structure and hence the swelling of the conjugates which in turn potentiates the use of corn Starch-Neusilin UFL2 as a tablet superdisintegrant [21].

#### 3.1.4. DSC Analysis

**Figure 3:**
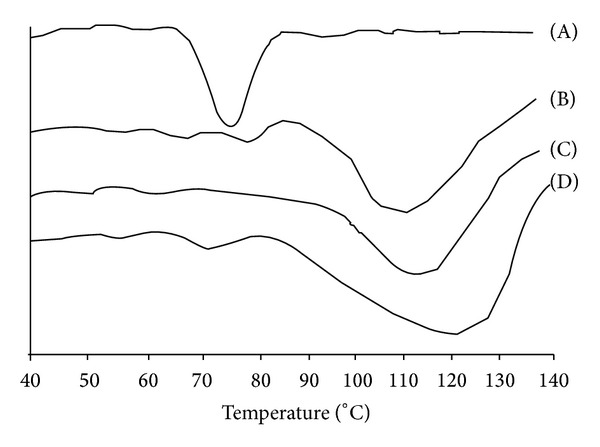
DSC pattern of (A) native corn starch; corn Starch-Neusilin UFL2 conjugates by (B) physical method, (C) chemical method, and (D) microwave method.

Figure 3 shows the DSC thermograms of the pure corn starch and corn Starch-Neusilin UFL2 conjugates prepared by physical, chemical, and microwave methods. Corn starch has a discrete structure and possesses partially crystalline microscopic granules that are held together by intended micellar network of associated molecules so it does not show obvious Tg or Tm until pyrogenation. Melting range was found to be extended and broadened with the incorporation of Neusilin UFL2. Broadening and shifting of the melting range in the DSC spectra could be attributed to intermolecular bonding between corn starch and Neusilin UFL2 indicating that the Neusilin UFL2 molecules were restrained by the corn starch molecules. The broadening of the melting range could be due to the regularity of the OH group that already existed in corn starch which had disappeared by the interaction with Neusilin UFL2. The Tm and ΔH of the melting peak are significantly lower in the pure corn starch than the conjugates which indicate that there is an interaction between corn starch and Neusilin UFL2 which has enhanced the crystallization of the pure corn starch. This increase in crystalline behaviour of the pure corn starch could be correlated to increase in swelling and hence superdisintegrant potential of conjugates prepared by different methods. Crystallinity and superdisintegrant behaviour of conjugates prepared by different methods could be arranged as microwave > chemical > physical mixture. Furthermore, the crystalline structure contributes towards the swelling behaviour of the conjugates, responsible for the tablet superdisintegrant activity.

#### 3.1.5. SEM Analysis

**Figure 4:**
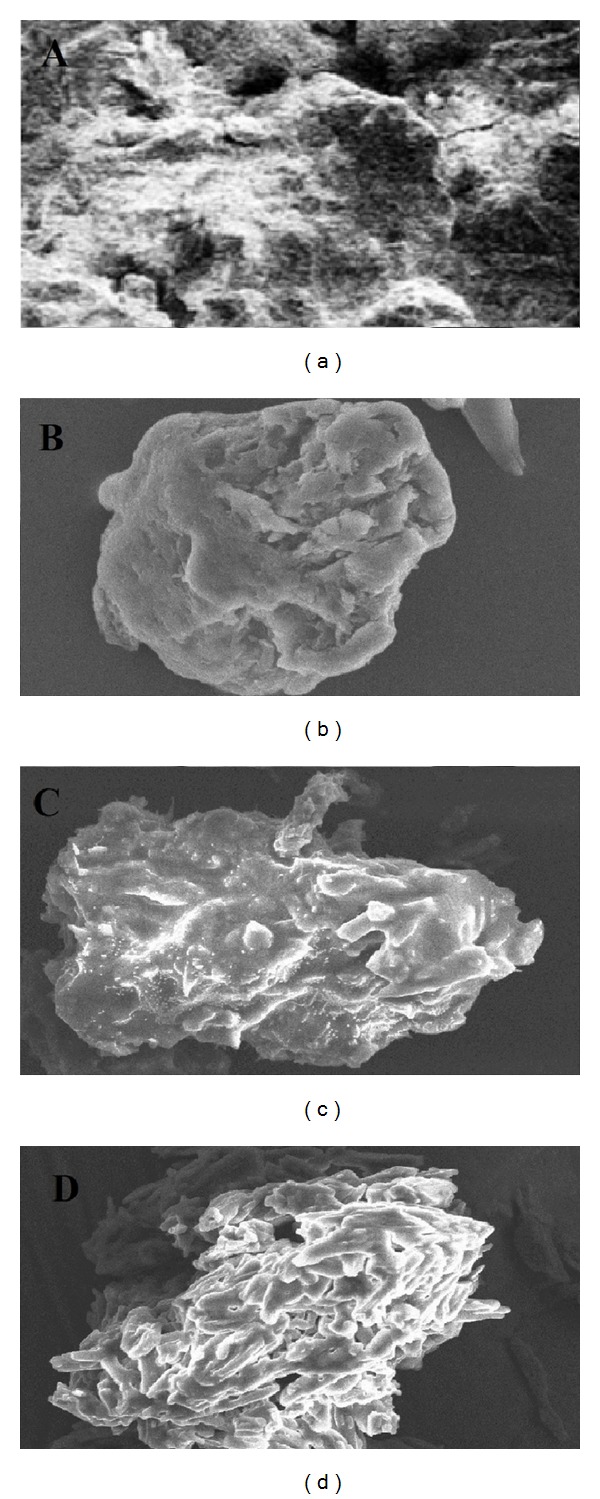
SEM photomicrographs of (A) corn starch and corn Starch-Neusilin UFL2 conjugates prepared by (B) physical method, (C) chemical method, and (D) microwave method.

Surface morphology of the corn starch and corn Starch-Neusilin UFL2 conjugates was studied by SEM analysis as shown in Figure 4. The corn starch undergoes a change in its native structure from thin smooth, flat surface structure, with folded edges, to three dimensional compacts upon conjugation with the Neusilin UFL2. SEM micrograph of the prepared conjugates showed the presence of interparticulate voids and channels that are responsible for the increase in water absorption and swelling capacity of the conjugates as compared to the pure corn starch. Furthermore these voids/channels contributed to the wicking behavior responsible for the tablet superdisintegrant property of the corn Starch-Neusilin UFL2 conjugates. Conjugates prepared by microwave method illustrated the presence of more porous structure as compared to conjugates prepared by physical and chemical methods. The results are in line with the better* in vitro* disintegration performance of FDT prepared with conjugates of microwave method compared to the other two methods.

#### 3.1.6. Heckel Function Analysis

**Figure 5:**
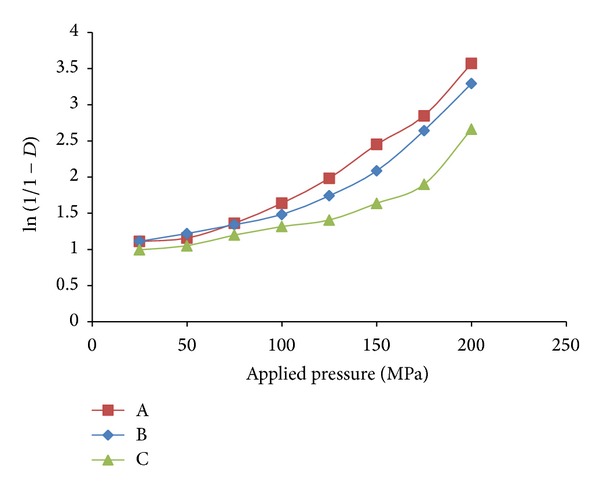
Heckel plots for the tablet incorporating corn Starch-Neusilin UFL2 conjugate as a superdisintegrant prepared by (A) physical method, (B) chemical method, and (C) microwave method.

Figure 5 shows representative Heckel plots for the conjugates prepared by physical, chemical, and microwave methods. The Heckel plots showed an initial linear portion with an increase slope at pressure of 100 MPa followed by another linear region for the conjugates prepared by physical and chemical methods while for the conjugate prepared by microwave method the second linear region was from 175 MPa. The mean yield pressure values for the conjugates were calculated from the slope of the portion showing the highest linearity of the Heckel plots, and the intercept, *A*, was determined from the extrapolation of the region. The values of *D*_*A*_ and *D*_*B*_ were calculated, respectively. The values of *P*_*y*_, *D*_0_, *D*_*A*_, and *D*_*B*_ for the formulations are presented in Table 3. The value of *D*_0_ which represents the degree of initial packing in the die, as a result of die filling for the conjugates, indicates that the conjugates prepared by microwave method exhibited highest degree of packing in the die as a result of die filling while the conjugates prepared by physical method exhibited the lowest values. The value of *D*_*B*_ represents the phase of rearrangement of the particles in the early stages of compression. *D*_*B*_ values tend to indicate the extent of fragmentation of particles or granules, although fragmentation can occur concurrently with plastic and elastic deformation of constituent particles. The chemically prepared conjugates exhibited the highest values while the one prepared by microwave method exhibited the lowest values. This indicates that fragmentation occurs more with the chemically prepared conjugates [22, 23]. The values of *D*_*A*_, which represents the total degree of packing achieved at zero and low pressures, was also in the rank order of microwave > chemical > physical for the methods to prepare conjugates. This indicates that the conjugates prepared by microwave method showed higher degree of packing at low pressures. The mean yield pressure *P*_*y*_ is inversely related to the ability of the formulations to deform plastically under pressure. The result indicates that the conjugates prepared by physical method showed the fastest onset of plastic deformation while the conjugates prepared by microwave method showed the slowest onset. Materials with high yield pressure are classified as brittle or fragmenting materials, whereas those with lower values are classified as plastically/elastically deforming materials [24]. Generally during compression, plastic deformation and fragmentation are known to occur concurrently. Starches have been known to deform plastically under compression pressure.

#### 3.1.7. Kawakita Function Analysis

**Figure 6:**
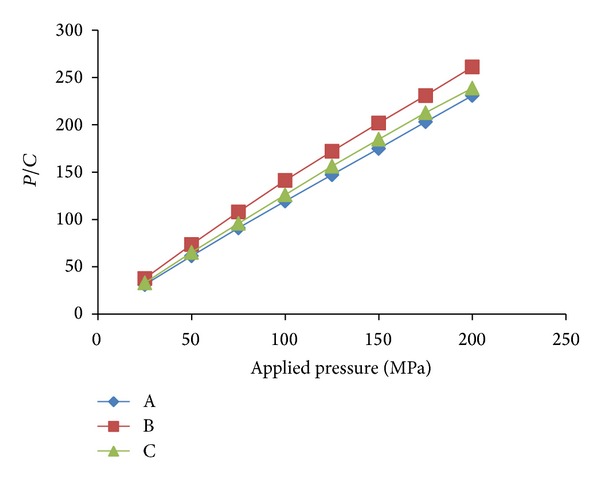
Kawakita plots for the tablet incorporating corn Starch-Neusilin UFL2 conjugate as a superdisintegrant prepared by (A) physical method, (B) chemical method, and (C) microwave method.

The Kawakita plots for the conjugates are presented in Figure 6. A linear relationship was obtained at all compression pressures employed with correlation coefficient of 0.999 for the conjugates prepared by different methods. The values of *a* and *ab* were obtained from the slope and intercept, respectively. The value of (1 − *a*) gives the initial relative density of the starch, *D*_*I*_, while *P*_*k*_ values were obtained from the reciprocal of values of *b*. The values of *D*_*I*_ and *P*_*k*_ are shown in Table 3. The value of *D*_*I*_ is a measure of the packed initial relative density of the formulation with the application of small pressure or tapping. The ranking of *D*_*I*_ for the conjugates was chemical method > microwave method > physical method. The value of *P*_*k*_ which is an inverse measure of the amount of plastic deformation occurring during the compression process was found to be maximum for tablets formulated using conjugates prepared by chemical method followed by microwave method and physical method [23]. Thus, conjugates prepared by microwave method exhibited the highest amount of total plastic deformation while conjugates prepared by physical method exhibited the lowest values. The ranking was seen to be in the reverse order as that of the *P*_*y*_ values. It has been shown that while *P*_*y*_ relates to the onset of plastic deformation during compression, the *P*_*k*_ relates to the amount of plastic deformation that occurs during the compression process. Thus, the conjugates prepared by physical method showed the slowest onset of plastic deformation but the highest total amount of plastic deformation.

### 3.2. Postcompression Evaluation

Tablets require certain amount of strength and resistance to friability to withstand mechanical shock of handling during manufacturing, shipping, and packaging. Hardness of the tablets formulated using the corn starch-Neusilin UFL2 conjugates as superdisintegrant was found to vary from 4.3 to 3.5 kg/cm^2^ compared to 3.15 to 3.09 kg/cm^2^ of tablets formulated using the native corn starch. Percentage friability of all formulations was less than 1% indicating good mechanical characteristics. Wetting time was observed to be decreased from 68 seconds for the tablets incorporating native corn starch to 33, 22, and 14 seconds for the tablets incorporating conjugates prepared by physical, chemical, and microwave methods. Disintegration time was also observed to decrease from 83 seconds for the tablets incorporating native corn starch to 40, 35, and 22 seconds for the tablets incorporating conjugates prepared by physical, chemical, and microwave methods, respectively. Water absorption ratio was found to be inversely proportional to the wetting and disintegrating time of the tablets. The increase in water absorption ratio and decrease in wetting and disintegration times in all formulations may be attributed to the modification of the corn starch by Neusilin UFL2 to yield a novel conjugate which acts as superdisintegrant which absorbs water and swells, causing rupture of the tablets. Tablet properties such as mechanical strength, tablet packing fraction, and disintegration are in turn affected by the porosity [25]. Tablets with low packing fraction have high porosity and pores facilitates the penetration of dissolution media into the tablet leading to disintegration of the tablet. Whereas higher tablet packing fraction leads to reduction in porosity which inhibits the penetration of dissolution media resulting in slower disintegration rate of the tablet [26]. Tablet packing fraction was found to be decreased in the tablets incorporating conjugates (0.63–0.41) compared to the tablets with corn starch (0.82). The results are in line with the powder evaluation results where conjugates prepared by physical, chemical, and microwave methods were showing better swelling and effective pore radius compared to the native corn starch. The fast disintegration of tablets is due to the presence of pores, resulting in faster penetration of the dissolution media, leading to swelling and wicking of the prepared conjugate, creating hydrodynamic pressure inside the tablets, hence responsible for the quick and complete disintegration of tablets indicating the superdisintegrant activity of conjugates prepared by physical, chemical, and microwave methods over the native corn starch.

**Figure 7:**
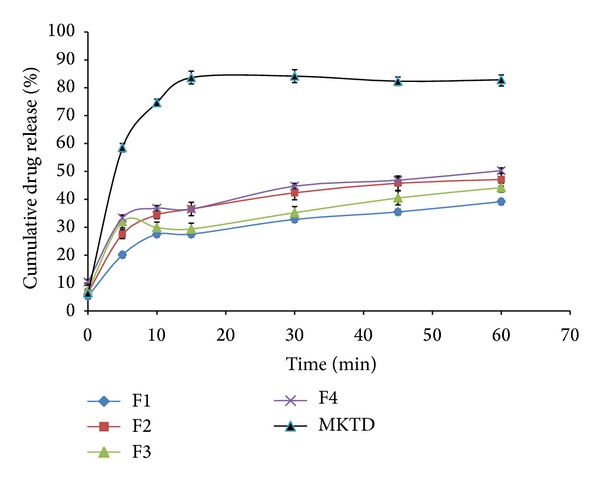
Dissolution rate profiles of FDTs (F1–F4) formulated by incorporating native corn starch as a superdisintegrant compared to a standard marketed formulation of domperidone (MKTD).

**Figure 8:**
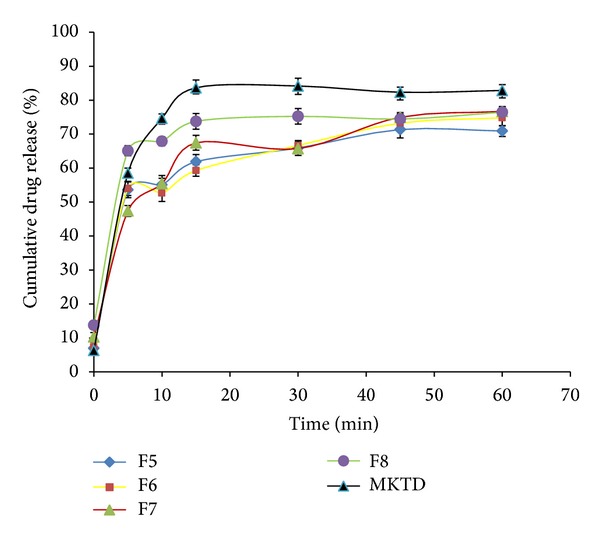
Dissolution rate profiles of FDTs (F5–F8) formulated by incorporating corn Starch-Neusilin UFL2 conjugates prepared by physical method as a superdisintegrant compared to a standard marketed formulation of domperidone (MKTD).

**Figure 9:**
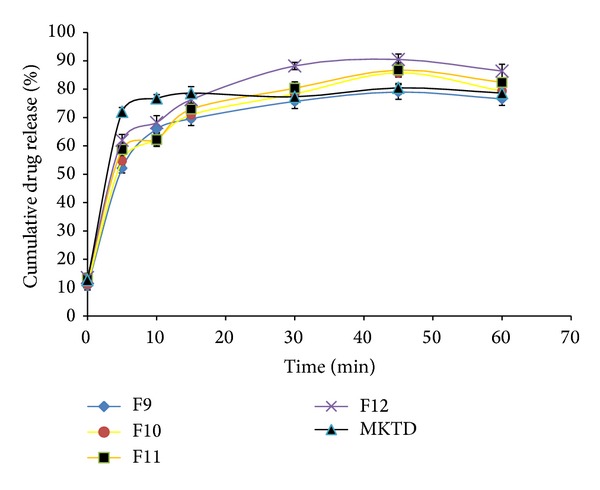
Dissolution rate profiles of FDTs (F9–F12) formulated by incorporating corn Starch-Neusilin UFL2 conjugates prepared by chemical method as a superdisintegrant compared to a standard marketed formulation of domperidone (MKTD).

**Figure 10:**
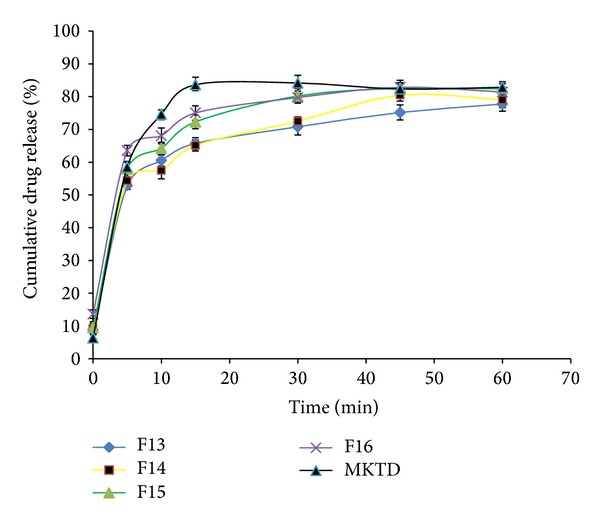
Dissolution rate profiles of FDTs (F13–F16) formulated by incorporating corn Starch-Neusilin UFL2 conjugates prepared by microwave method as a superdisintegrant compared to a standard marketed formulation of domperidone (MKTD).

The results obtained from the* in vitro* dissolution study are presented in Figures 7, 8, 9, and 10. “MKTD” represents the standard marketed domperidone formulation to which the formulated FDTs were compared. The similarity factor (*f*_2_) is a logarithmic transformation of the sum-squared error of differences between test and reference products over all time points. It was observed that the three different approaches used for the preparation of conjugate in this study, namely, physical, chemical, and microwave methods, increased the dissolution rate of the drug compared to the tablet prepared with the native corn starch and standard marketed formulation of the drug. The order of increased drug dissolution using the different approaches was as follows: microwave > chemical > physical. In case of the FDTs prepared by using native corn starch (F1–F4) the percentage cumulative drug release was found to be very poor as compared to the standard marketed formulation of domperidone (MKTD). The *f*_2_ value for formulation (F1–F4) was below 50 depicting the dissimilarity between the drug releases. Formulations (F5–F8), (F9–F12), and (F13–F16) were containing corn starch-Neusilin UFL2 conjugate prepared by physical, chemical, and microwave methods. The percentage cumulative drug release for these formulations were found to be comparable to the standard marketed formulation of domperidone. The *f*_2_ values for these formulations were found to be over 50 evidencing the similarity between the drug releases. Results of various tablet evaluation tests as evidenced from Table 4, indicate that among the three methods used for the preparation of conjugates, microwave was the most effective method followed by chemical and physical methods. Moreover by increasing the concentration of conjugates, the tablet did not show much effect on drug release from the formulated FDTs. Hence from the commercial point of view, lowest concentration of superdisintegrant, showing optimum tabletting results, should be recommended.

## 4. Conculsions

In present work, an attempt was made to modify the corn starch to develop corn starch-Neusilin UFL2 conjugates using different methods, namely physical, chemical, and microwave. The prepared conjugates and the corn starch were characterised for powder flow, pH, viscosity, swelling, and effective pore radius. Evaluation of precompression parameter indicates good flow properties. The prepared conjugates were also subjected to the compression studies, namely Heckel and Kawakita function. The Heckel plot is usually correlated with the speed of the tablet machine while the Kawakita plot is related to the crushing or tensile strength of the tablet. Fast disintegrating tablets of domperidone were formulated by direct compression technique employing the corn starch and the prepared conjugates. The tablets were evaluated for physical parametric tests, wetting time, water absorption ratio, porosity, tablet packing fraction,* in vitro* disintegration time, drug content,* in vitro* dissolution, and stability studies. Extensive swelling, porosity, and wicking action of the prepared corn Starch-Neusilin UFL2 conjugates in the prepared fast disintegrating tablets were found to be contributing to its superdisintegrant action.

In conclusion, the prepared conjugates can be effectively used as superdisintegrant in order to develop a faster disintegrating tablet formulation. Future challenges for many fast disintegrating tablet manufacturers include reducing costs by finding ways to manufacture, with conventional equipment, using versatile packaging, and improving mechanical strength. Such products provide an opportunity for the product line extension in the market place and extension of patent term of innovator. Due to its wide significance, this drug delivery system may lead to better patient compliance and ultimate clinical output. Future might witness novel technologies for development and utilization of the modified starches as tablet superdisintegrant for cost effective formulation of fast disintegrating tablets.

## Figures and Tables

**Table 1:** Formulation table for preparing FDTs.

Code	Ingredients (mg)								
	Domperidone	Native corn starch	Corn Starch-Neusilin UFL2 conjugate			Avicel 102	Magnesium stearate	Talc	TW∗
			Physical mixture	Chemical mixture	Microwave mixture				
F1	10	2.5	—	—	—	85.5	1	1	100
F2	10	5	—	—	—	83	1	1	100
F3	10	7.5	—	—	—	80.5	1	1	100
F4	10	10	—	—	—	78	1	1	100
F5	10	—	2.5	—	—	85.5	1	1	100
F6	10	—	5	—	—	83	1	1	100
F7	10	—	7.5	—	—	80.5	1	1	100
F8	10	—	10	—	—	78	1	1	100
F9	10	—	—	2.5	—	85.5	1	1	100
F10	10	—	—	5	—	83	1	1	100
F11	10	—	—	7.5	—	80.5	1	1	100
F12	10	—	—	10	—	78	1	1	100
F13	10	—	—	—	2.5	85.5	1	1	100
F14	10	—	—	—	5	83	1	1	100
F15	10	—	—	—	7.5	80.5	1	1	100
F16	10	—	—	—	10	78	1	1	100

TW∗: total weight of tablet in mg.

**Table 2:** Different powder properties.

Serial number	Parameter	Observation			
		Native corn starch	Corn Starch-Neusilin UFL2 conjugate		
			Physical mixture	Chemical mixture	Microwave mixture
1	Bulk density (g/cm^3^)	0.46 ± 0.06	0.31 ± 0.04	0.33 ± 0.06	0.28 ± 0.05
2	Tapped density (g/cm^3^)	0.70 ± 0.01	0.40 ± 0.09	0.41 ± 0.02	0.32 ± 0.07
3	Carr's index (%)	34.28 ± 0.12	22.5 ± 0.17	19.51 ± 0.10	12.5 ± 0.09
4	Hausner ratio	1.52 ± 0.04	1.29 ± 0.08	1.24 ± 0.04	1.14 ± 0.05
5	Angle of repose (*θ*°)	43.25 ± 0.34	36.69 ± 1.04	31.88 ± 0.95	27.67 ± 1.82
6	Swelling index (%)	18	40	65	95
7	pH	6	8	8	8
8	LOD (%)	10.08 ± 0.09	8.40 ± 0.12	6.79 ± 0.26	4.16 ± 0.22
9	Effective pore radius (mm)	11.45 ± 0.27	18.26 ± 0.36	22.35 ± 0.25	29.14 ± 0.22

**Table 3:** Parameters derived from the Heckel and Kawakita plots for tablet incorporating corn Starch-Neusilin UFL2 conjugate as a superdisintegrant prepared by (A) physical method, (B) chemical method, and (C) microwave method.

Sample	Heckel analysis				Kawakita analysis	
	*D*_0_	*D*_*A*_	*D*_*B*_	*P*_*y*_	*D*_*I*_	*P*_*k*_
A	0.179	0.288	0.108	52.91	0.120	3.88
B	0.295	0.373	0.078	55.25	0.211	8.29
C	0.365	0.538	0.178	172.41	0.151	5.39

**Table 4:** Different properties of the formulated FDTs.

Code	Parameter											
	Diameter (mm)	Thickness (mm)	Friability (%)	Hardness (kg/cm^2^)	TS∗ (MN/m^2^)	WT∗ (Sec)	WAR∗ (%)	DT∗ (Sec)	CU∗ (%)	TPF∗	P* (%)	*F*_2_
F1	6.74 ± 0.03	3.56 ± 0.03	0.89 ± 0.04	3.11 ± 0.10	0.47 ± 0.15	68 ± 1.13	40 ± 0.11	73 ± 3	98.99 ± 0.2	0.81	18.9	19
F2	6.73 ± 0.04	3.51 ± 0.04	0.91 ± 0.02	3.15 ± 0.07	0.47 ± 0.05	68 ± 1.29	42 ± 0.11	73 ± 1	99.1 ± 0.15	0.82	17.5	24
F3	6.74 ± 0.05	3.55 ± 0.05	0.89 ± 0.05	3.09 ± 0.11	0.47 ± 0.11	67 ± 1.11	46 ± 0.09	71 ± 2	97.27 ± 0.9	0.81	18.7	21
F4	6.73 ± 0.01	3.59 ± 0.05	0.90 ± 0.03	3.10 ± 0.02	0.47 ± 0.09	66 ± 1.14	46 ± 0.10	71 ± 3	96.59 ± 0.5	0.80	19.4	25
F5	6.74 ± 0.01	3.51 ± 0.05	0.76 ± 0.04	3.50 ± 0.11	0.48 ± 0.05	33 ± 1.14	98 ± 0.04	48 ± 2	96.50 ± 0.3	0.63	36.7	43
F6	6.74 ± 0.02	3.55 ± 0.04	0.73 ± 0.05	3.56 ± 0.08	0.48 ± 0.01	33 ± 1.67	96 ± 0.04	46 ± 3	99.1 ± 0.15	0.62	37.3	43
F7	6.74 ± 0.01	3.55 ± 0.05	0.71 ± 0.02	3.70 ± 0.15	0.56 ± 0.18	32 ± 1.10	101 ± 0.05	44 ± 1	98.35 ± 0.2	0.62	37.3	45
F8	6.74 ± 0.03	3.56 ± 0.03	0.67 ± 0.03	3.75 ± 0.21	0.55 ± 0.08	31 ± 1.40	103 ± 0.02	40 ± 2	99.23 ± 0.5	0.62	37.5	47
F9	6.73 ± 0.04	3.55 ± 0.02	0.55 ± 0.01	4.00 ± 0.18	0.60 ± 0.19	23 ± 1.34	108 ± 0.03	40 ± 6	99.12 ± 0.4	0.54	42.6	50
F10	6.74 ± 0.05	3.54 ± 0.07	0.54 ± 0.05	3.80 ± 0.15	0.57 ± 0.06	22 ± 1.25	100 ± 0.02	35 ± 3	98.92 ± 0.7	0.57	43.5	50
F11	6.73 ± 0.03	3.56 ± 0.04	0.55 ± 0.03	3.70 ± 0.17	0.56 ± 0.11	23 ± 1.05	106 ± 0.06	36 ± 2	97.27 ± 0.9	0.57	42.3	55
F12	6.73 ± 0.02	3.53 ± 0.03	0.51 ± 0.04	4.10 ± 0.2	0.62 ± 0.15	23 ± 1.38	110 ± 0.04	32 ± 7	96.59 ± 0.5	0.56	42.6	53
F13	6.74 ± 0.03	3.55 ± 0.04	0.41 ± 0.07	4.20 ± 0.27	0.73 ± 0.08	14 ± 1.52	121 ± 0.08	26 ± 2	99.15 ± 0.1	0.41	58.2	50
F14	6.73 ± 0.01	3.51 ± 0.04	0.42 ± 0.02	4.25 ± 0.2	0.73 ± 0.20	14 ± 1.26	124 ± 0.07	24 ± 5	98.99 ± 0.2	0.39	59.5	52
F15	6.74 ± 0.03	3.55 ± 0.06	0.44 ± 0.04	4.30 ± 0.10	0.76 ± 0.18	13 ± 1.15	125 ± 0.03	22 ± 3	99.75 ± 1.0	0.40	59.9	62
F16	6.74 ± 0.01	3.59 ± 0.05	0.47 ± 0.07	4.20 ± 0.45	0.79 ± 0.19	14 ± 1.40	128 ± 0.05	22 ± 4	99.12 ± 0.3	0.41	61.2	64

TS∗: tensile strength; WT∗: wetting time; WAR∗: water absorption ratio; DT∗: disintegration time; CU∗: content uniformity; TPF∗: tablet packing fraction; *P*∗: porosity.
